# First Study of *Ascaris lumbricoides* from the Semiwild Population of the Sumatran Orangutan *Pongo abelii* in the Context of Morphological Description and Molecular Phylogeny

**DOI:** 10.3390/life13041016

**Published:** 2023-04-14

**Authors:** Kristína Civáňová Křížová, Mária Seifertová, Vlastimil Baruš, Iveta Hodová, Šárka Mašová, Wisnu Nurcahyo, Ivona Foitová

**Affiliations:** 1 Department of Botany and Zoology, Faculty of Sciences, Masaryk University, Kotlářská 2, 611 37 Brno, Czech Republic; 2 Department of Parasitology, Faculty of Veterinary Medicine, Gadjah Mada University, Jl. Fauna 2, Yogyakarta 55281, Indonesia

**Keywords:** Sumatran orangutan *Pongo abelii*, *Ascaris lumbricoides*, ascarids, non-human primates, phylogeny reconstruction, species determination, cytochrome C oxidase I (*CO1*), internal transcribed spacer (*ITS*), host switching

## Abstract

There is little evidence that the already described and accepted taxa of ascarids (*Ascaris lumbricoides*, *A. suum*, and *A. ovis*) infecting individuals of taxonomically distant groups (hominids, pigs, sheep, goats, and dogs) can be genetically or morphologically distinguished. However, despite described morphological differences, e.g., due to intraspecific variation, these are insufficient for species determination and may indicate differences amongst ascarids because of cross infections, hybrid production, and specific adaptations to hosts. Herein, the results of a molecular and morphological analysis of ascarids parasitising Sumatran orangutans (*Pongo abelii* Lesson, 1827) in native populations are presented. The research took place in the Bukit Lawang area, Indonesia, in 2009. Throughout the year, fresh faecal samples were collected regularly from 24 orangutans, and all were examined for the presence of nematode adults. Only five adult worms from two orangutan females were found during regular collection. Using the integrative taxonomic approach, the nematodes found were identified as *A. lumbricoides*. The significance of the find and its rarity is documented by the fact that this is the first confirmed finding of adult ascarids from an original orangutan site (not from a zoo) in more than 130 years (including the long-term study spanning the last 20 years focusing on orangutan parasites and natural antiparasitic drugs). More accurate morphometric parameters and genetic differences for the identification of ascarids were established. These parameters will be helpful for other findings in great apes and will also be suitable for further and precise determination of this parasite. The details distinguishing between male and female specimens are also stated and well defined. A comprehensive evaluation of the situation of *Ascaris* species parasitising orangutans, including a comparison with previously described orangutan parasite (i.e., *A. satyri*—species inquirenda), is discussed.

## 1. Introduction

Orangutans (genus *Pongo* Lacépède, 1799, Hominidae) are the only great ape species found exclusively in Asia [1] and are recognised as Critically Endangered by the IUCN [2]. The health of orangutans can be affected by a wide variety of intestinal parasites, among which nematodes play a significant role, as they have the potential to cause numerous serious health problems [3,4]. Because of this, the accurate identification of nematode species parasitising orangutans—including the genus *Ascaris* Linnaeus, 1758—has become increasingly important in recent decades [5,6,7,8,9].

According to the literature on ascarid findings in orangutans (Table 1), not considering *Ascaris* sp. and *Ascaris* spp., only two species, namely *A. lumbricoides* (Linnaeus, 1758) and *A. satyri* Chatin, 1877, have been consistently found. Regarded as a typically cosmopolitan parasite of humans, *A. lumbricoides* findings from primates, especially from great apes, have been very rare. However, records of this species have increased in the last two decades [7,9,10,11,12,13,14]. The species *A. satyri* is regarded by the author of its original description as a specialised parasite of Bornean orangutans. The name of this taxon is mentioned in a checklist by Linstow [15], but it is missing in modern monographs and is not mentioned even as a synonymic name of *A. lumbricoides* in some older literature [16,17,18]. Only Sprent in 1968 states the name of *A. satyri* with the remark that the inadequacy of the description prevented its inclusion in the genus *Ascaris* [19]. Later, Kirby with colleagues in 1975 mentioned this species as excluded from the genus *Ascaris* [20].

Ascarids parasitising orangutans are known mainly from their descriptions in captive hosts (Table 1). All reported ascarids from hosts living in native zoogeographical areas in Kalimantan (Borneo) or Sumatra have been based strictly on coprological analyses and on measurements of egg size; no molecular analyses have been used. The ascarids identified using these methods were all either *A. lumbricoides* [21,22,23] or *Ascaris* sp. [7,8,24,25,26]. Ascarids found in orangutans have not yet been morphologically studied, documented, or determined in detail.

This is the first study to present the results of morphological and molecular-genetic research on ascarids found in Sumatran orangutans from native populations. It comprehensively proves the ability of nematode *Ascaris* spp. to parasitise orangutans as well as different unrelated vertebrates.

**Table 1:** List of *Ascaris* species reported from host of the genus *Pongo* Lacépède, 1799.

Species	Host	Material	Locality	References
*A.lumbricoides*	*P. pygmaeus*, captive	specimens	France Zoo	[27]
*A.satyri*	*P. pygmaeus*, captive	specimens	Museum Paris (imported from Borneo)	[28]
*A.lumbricoides*	*P. pygmaeus*, captive	specimens	Zoo Calcutta (India)	[29]
*A.lumbricoides*	*P. pygmaeus*, captive	specimens	Zoo Philadelphia (imported from Borneo)	[30]
*A.lumbricoides*	*P. pygmaeus*, captive	specimens	Zoo (Japan)	[31]
*A.lumbricoides*	*P. pygmaeus*, captive	specimens	Zoo London (UK)	[32,33]
*A.lumbricoides*	*P. pygmaeus*, captive	specimens	Zoo Amsterdam Rotterdam	[34]
*Ascaris* spp.	*P. pygmaeus*, captive	eggs	Primate Center Atlanta (USA)	[35]
*Ascaris* sp.	*Pongo* spp. captive, semi-wild	eggs	Zoo Jakarta, Sumatra-Bohorok, Kalimantan-Tanjung Puting (Indonesia)	[26]
*A.lumbricoides*	*P. abelii*, wild	Just simple list	Sumatra-Ketambe (Indonesia)	[36]
*Ascaris* sp.	*P. pygmaeus*, rehabilitant	eggs	Kalimantan-Tanjung Puting (Indonesia)	[24]
*Ascaris* sp.	*P. pygmaeus*, captive	eggs	Zoo Jakarta (Indonesia)	[24]
*Ascaris* sp.	*P.abelii*, rehabilitant	eggs	Sumatra-Bohorok (Indonesia)	[24]
“Ascarids”	*Pongo* spp. captive	eggs and specimens	from 50 international Zoo institutions	[37]
*Ascaris* sp.	*P. abelii*, wild	eggs	Sumatra-Ketambe (Indonesia)	[25]
*A.lumbricoides*	*P. pygmaeus*, during rehabilitation	eggs	Kalimantan-Tanjung Puting (Indonesia)	[21]
*Ascaris* sp.	*P. pygmaeus*, captive, semi-wild	eggs	Kalimantan-Wanariset (Indonesia)	[38]
*A. lumbricoides*	*P. abelii*, semi-wild	eggs	Sumatra-Bohorok (Indonesia)	[22]
*A. lumbricoides*	*P. pygmaeus*, captive	eggs	Zoo Wroclaw (Poland)	[39]
*Ascaris* sp.	*P. abelii*, captive	eggs	Sumatra (Indonesia)	[8]
*Ascaris* sp.	*P. abelii*, captive	eggs	Sumatra-quarantine Batu Mbelin (Indonesia)	[40]
*Ascaris* sp.	*Pongo* spp., captive	eggs	Zoo Rizal (Philippines)	[41]
*Ascaris* sp.	*P. pygmaeus*, captive	eggs	Kalimantan-Nyaru Menteng, Wanariset (Indonesia)	[7]
*A. lumbricoides*	*P. pygmaeus*, *wild*	eggs	Kalimantan-Sebangau (Indonesia)	[23]
*Ascaris* spp.	*P. pygmaeus*, *wild*, *urban*, *captive*	eggs	Peninsular Malaysia and Malaysian Borneo (Sabah and Sarawak)	[10]
*Ascaris* sp.	*P. pygmaeus*, *captive*	eggs	Matang Wildlife Centre, Kuching Division, Sarawak (Malaysia)	[9]

## 2. Methods

### 2.1. Sample Collection

The location of this study, the village of Bukit Lawang (the former site of a rehabilitation centre for Sumatran orangutans), is situated on the southwest border of the Gunung Leuser National Park, North Sumatra, Indonesia (N 03°32.983′ E 098°06.908′), at an altitude of 323 m. Temperatures in this region range between 21 °C and 28 °C, with humidity levels ranging between 80% and 100%. Annual rainfall ranges from 2000 mm to 3200 mm.

The majority of Sumatran orangutans (*Pongo abelii* Lesson, 1827) in this area belong to a semi-wild population. At the time of this study (2009), nine orangutans were in cages in the quarantine area and fifteen orangutans were roaming freely within the vicinity of the quarantine area, so contact between these individuals was possible. Continuously throughout the whole year, the faecal samples were collected regularly and examined for the presence of nematode adults. In total, five (*n* = 5) adult specimens were found from the fresh faeces of two semi-wild adult females and immediately fixed in 70% ethanol.

### 2.2. Morphological Analyses

For microscopic examination, sections of the nematode body were cleared gradually in glycerine and examined for morphometric analysis under a light microscope (Olympus BX51) equipped with differential interference contrast (DIC), a digital image analysis system (Micro Image 4.0 for Windows), and a drawing attachment. All provided measurements are given in micrometers (µm) unless stated otherwise, and are given as the range followed by the mean in parentheses. The parasite specimen (one female, anterior part of the body) selected for scanning electron microscopy (SEM) was further dehydrated in ethanol, dried in a CPD 030 critical point drying apparatus (Bal-tec) using liquid CO_2_, mounted on aluminium stubs with a double-sided adhesive disc, coated with gold in a SCD 040 sputter coating unit (Balzers), and examined in a MIRA (Tescan) scanning electron microscope operating at 15 kV.

### 2.3. DNA Extraction, PCR Amplification, and Gene Characterisation

Five adult worms collected from two orangutans in Sumatra were processed for molecular analyses. A middle part of the body of approximately 10–12 mm in length was cut off and fixed in 96% ethanol. In addition, one ascarid specimen collected from domestic pigs (*Sus scrofa domesticus* Erxleben, 1777) in the Czech Republic and one specimen of *Baylisascaris columnaris* (Leidy, 1856) obtained from the captive striped skunk (*Mephitis mephitis* (Schreber, 1776)) in the Czech Republic were also analysed, and obtained sequences were included into phylogenetic analyses.

Prior to DNA extraction, parasites were dried, and genomic DNA was isolated from each specimen using a DNeasy Blood and Tissue Kit (Qiagen, Hilden, Germany) following the recommended protocol with slight modification (overnight incubation at room temperature during lysis with proteinase K until tissues were fully digested). For molecular characterisation, partial sequences of one mitochondrial gene (*CO1*) and four nuclear regions for rRNA genes (*18S*, *5.8S*, *28S* and both internal transcribe spacers (*ITS1*, *ITS2*)) were amplified by using the primer sets listed in Table 2. Each polymerase chain reaction (PCR) was performed in 30 μL of reaction volume using 20–50 ng of genomic DNA as a template. For amplification of the *CO1* fragment, a PCR reaction mixture consisted of 1× PPP Master Mix (Top-Bio, Vestec, Czech Republic), 0.3 μM of each primer, template DNA in a stated concentration, and nuclease-free water. Amplification ran under the following conditions: initial denaturation at 95 °C for 5 min, then 35 cycles of 95 °C for 30 s, 55 °C for 30 s, and 72 °C for 40 s; a final extension ran at 72 °C for 10 min. The reaction mixture for *18S* rDNA amplification was comprised of DNA template, nuclease-free water, 1× *Taq* buffer with KCl, 1.5 mM MgCl_2_, 0.2 mM of each dNTP, 0.5 μM of each primer, and 1 U of *Taq* DNA polymerase (Fermentas by Thermo Fisher Scientific, Waltham, MA, USA, hereinafter Fermentas). Amplification ran in 39 cycles under the following conditions: initial denaturation at 94 °C for 5 min; cycling at 94 °C for 30 s, 54 °C for 30 s, and 72 °C for 1 min; a final extension ran at 72 °C for 10 min. Amplification of the *18S-ITS1-5.8S-ITS2-28S* region (referred to below as *ITS* only) was performed using four primer combinations: (1) TW81–AB28 [42]; (2) 18SNemF–28SNemR [43]; (3) ITS2-F–ITS2-R [44]; and (4) Ascaris ITS F1–Ascaris ITSR1 [45] (for details, see Table 2). Each reaction mixture was comprised of DNA template, nuclease-free water, 1× *Taq* buffer with KCl, 2 mM MgCl_2_, 0.2 mM of each dNTP, 0.5 μM of each primer, and 2 U of *Taq* DNA polymerase (Fermentas). Cycling parameters started with 5 min denaturation at 94 °C, then 39 cycles of 94 °C for 40 s, AT °C (Table 2) for 40 s, 72 °C for 1 min, and a final extension of 10 min at 72 °C. For TW81–AB28 primer combination, the elongation at 72 °C in cycling was prolonged up to 2 min. All PCR reactions were performed in a Mastercycler EP gradient S thermocycler (Eppendorf, Hamburg, Germany). The quality and yield of isolates, as well as PCR products, were verified via agarose gel electrophoresis on 1.5% gel stained with GoodView dye (SBS Genetech, Beijing, China).

For automatic sequencing, the obtained PCR products were purified according to manufacturers’ recommendations using the High Pure PCR Product Purification Kit (Roche, Basel, Switzerland) or enzymatic approach with ExoSAP-IT PCR cleanup reagent (Thermo Fisher Scientific, Waltham, MA, USA). Amplification was performed using the fluorescent chemistry of BigDye Terminator v3.1 Ready Reaction Cycle Sequencing Kit (Applied Biosystems by Life Technologies, Carlsbad, CA, USA; hereinafter Applied Biosystems). Subsequently, the products of the sequencing reactions were purified using BigDye X-Terminator Purification Kit (Applied Biosystems) according to the manufacturer’s protocol and sequenced in both directions using PCR primers on an ABI 3130 Genetic Analyzer (Applied Biosystems) under the appropriate run module. Raw sequencing data were analysed with Sequencing Analysis software v.5.2 (Applied Biosystems) and subjected to processing via Sequencer v.5.4.6 software (Gene Codes Corporation, MI, USA) to obtain contigs. The obtained sequences were deposited in GenBank database (IDs: LN600399–LN600409, and OQ539679–OQ539680–OQ539681). The Basic Local Alignment Search Tool (BLAST; https://blast.ncbi.nlm.nih.gov/Blast.cgi; accessed on 16 February 2023) searches were performed to verify the similarity of the sequences obtained in this study with other sequences of ascarid nematodes.

### 2.4. Sequence Alignment and Phylogenetic Analyses

Two sequence alignments were built to assess the phylogenetic placement of newly obtained sequences within Ascarididae. The first *CO1* alignment included one sequence of *Ascaris lumbricoides* isolated from *P. abelii* in Sumatra, 23 sequences representing the most common haplotypes of *Ascaris* species, and 13 sequences of other representatives of the family Ascarididae (details and accession numbers are stated in Table 3). According to previous published studies [48,49,50,51] and the GenBank/BLAST data search, the consensus haplotype sequences (i.e., HapA1-7, HapB1-4, and HapC1-4) were generated for analysis purposes for this study. The *CO1* sequences from these study (LN600399–LN600401) were trimmed from primer sequences before alignment. As outgroup, the sequences of four *Toxocara* species (AJ9220055; AJ920057; AJ920062; AM412316) were used. The second ITS alignment comprised three sequences generated in the present study (access. no. OQ539679–*A. lumbricoides* from *P. abelli*; OQ539680–*A. lumbricoides* from S*. scrofa domesticus* and OQ539681–*B. columnaris* from striped skunk) and 24 sequences downloaded from Genbank representing *Ascaris* species isolated from different host and selected representatives of Ascarididae (for details, sequence origin, and accession numbers see Figure 5). The sequences containing partial regions of *18S* and *28S* were trimmed before alignment to the *ITS* region only. Two sequences of *Toxocara* species (JF837169 and JF837171) were used as outgroup.

Sequences in both datasets were aligned in MAFFT v.7 [54,55] with default parameters and manually edited in BioEdit [56]. For the *CO1* sequences, the presence of stop codons and indels was verified with MEGA 11 [57], using the invertebrate mitochondrial code. Highly variable regions, extensive gaps, and poorly aligned positions present in the ITS alignment were eliminated using Gblocks 0.91b [58], allowing all options for a less stringent selection. Phylogenetic relationships were analysed using Bayesian inference (BI) and Maximum likelihood (ML) approaches. The best fitting models of nucleotide substitution were determined via ModelFinder [59] for each gene dataset. According to the Bayesian information criterion (BIC), the TIM3 + F + G model was selected as the most appropriate evolutionary model for CO1 dataset and K2P + G4 for ITS1, K2P + I for 5.8S, and HKY + F + G4 for ITS2 dataset. BI analysis was conducted using MrBayes 3.2.6 [60]. Two simultaneous runs with four independent Markov chains were performed for 5 million generations, sampling every 100 generations. After the average standard deviation of split frequencies fell below 0.01, the first 25% of samples were discarded as burn-in, and the remaining trees were used to generate a consensus tree and calculate the posterior probabilities. The ML analysis was performed in IQ-TREE v.2 [61]. To obtain node support statistics, 1000 bootstrap replicates using ultrafast bootstrap approximation (UFBoot) [62] and the Shimodaira–Hasegawa approximate likelihood ratio test (1000 replicates) were chosen. The obtained trees for BL and ML were visualised in FigTree v.1.4.3 [63].

### 2.5. Ethical Note

All the research reported in this manuscript adhered to the legal requirements of the country in which the work took place (SIP No 0056/FRP/SM/III/2009, 0008/EXT/FRP/SM/II/2010) CITES permit 08411/IV/SATS-LN2011). Since the collection of faecal samples from orangutans was non-invasive and did not involve interaction with or distress to the animals, the study was not reviewed by an animal ethics committee. No interaction with animals was conducted for this study.

## 3. Results

### 3.1. Morphological Analysis

**Figure 1:**
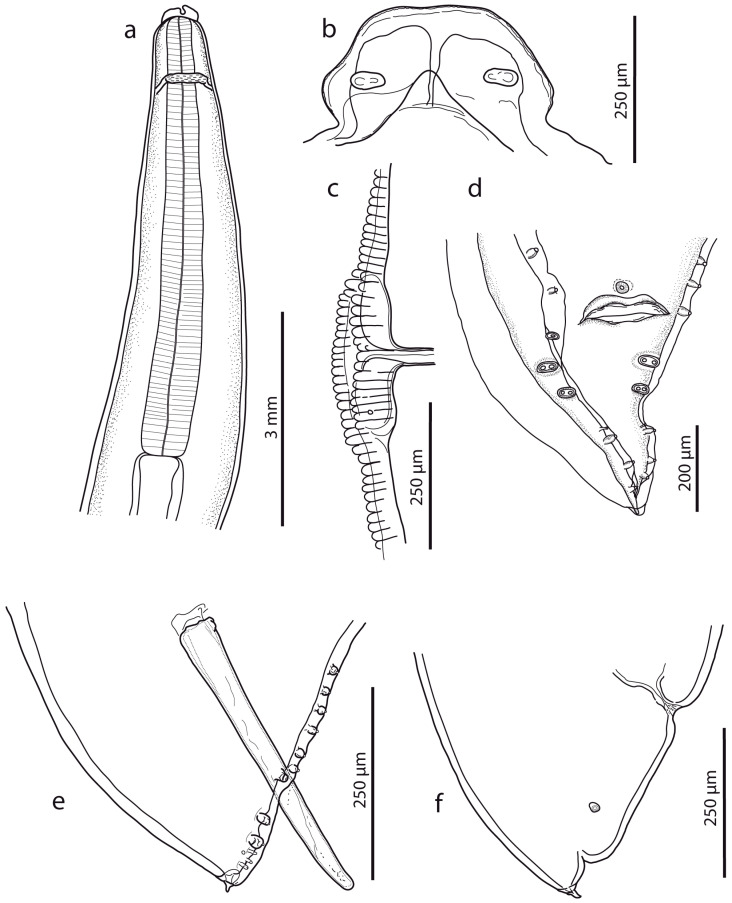
*Ascaris lumbricoides*, line drawings. (**a**) Anterior end with oesophageal region (lateral view). (**b**) Dorsal lip (detail). (**c**) Vulval region (lateral view). (**d**) Female caudal end (lateral view). (**e**) Male caudal end with papillae (ventral view) (for comparison, see [16]). (**f**) Male caudal end with papillae and spicule (lateral view).

**Figure 2:**
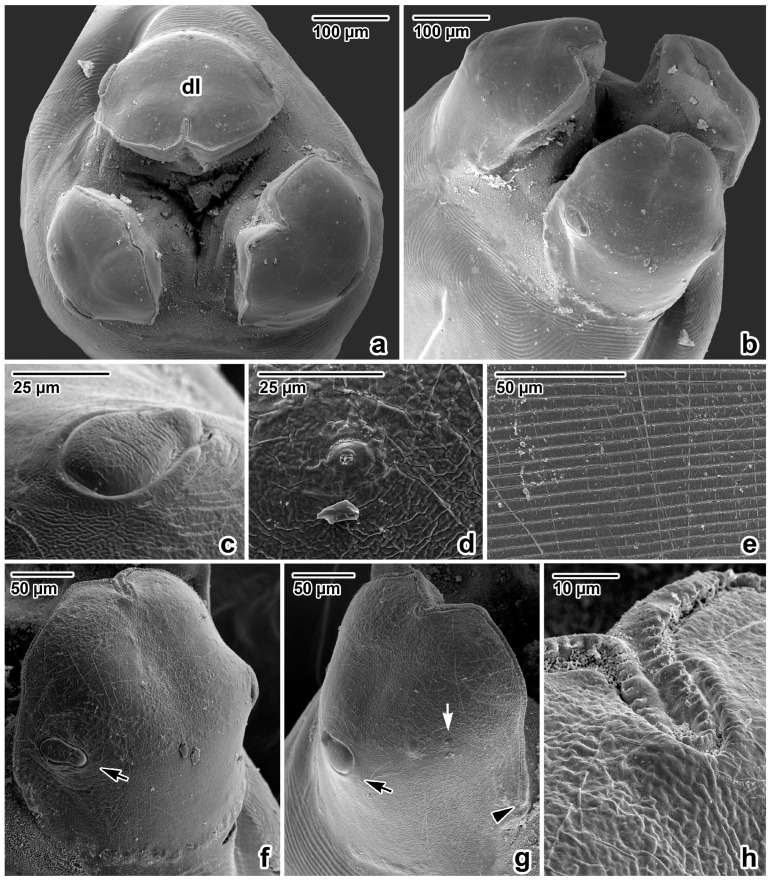
Anterior extremity of *Ascaris lumbricoides*, female, SEM micrographs. (**a**) Anterior extremity, apical view. (**b**) Anterior extremity, dorsolateral view. (**c**) Detail of the dorsal labial papilla. (**d**) Detail of the amphidial pore in lateroventral lip. (**e**) Detail of the cuticle, transverse striation in oesophageal part of body. (**f**) Dorsal lip, total view. (**g**) Lateroventral lip, total view. (**h**) Inflexed line of denticles in anterior extremity of dorsal lip (detail); *dl*—dorsal lip; *arrow*—papilla; *white arrow*—amphidal pore; *arrowhead*—end of denticle row.

**Figure 3:**
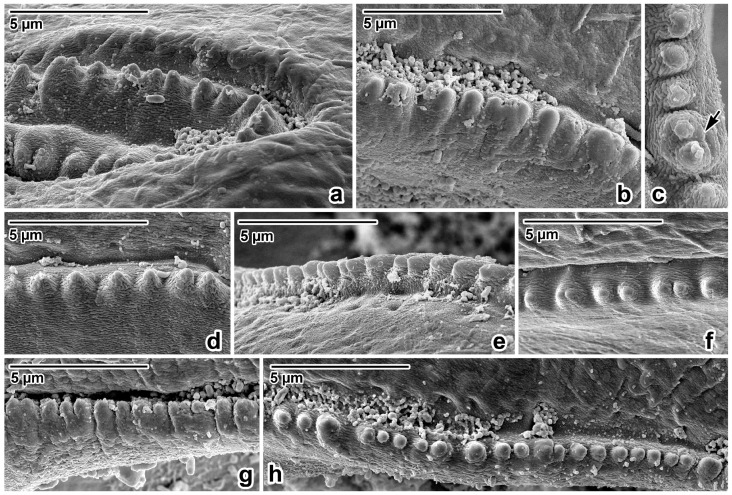
Lip rim details of *Ascaris lumbricoides*, female, SEM micrographs. (**a**,**b**) Anterior part of lip rim, lateral view. (**c**) Denticle with double peak, apical view. (**d**,**e**) Lateral part of lip rim, lateral view. (**f**) Lateral part of lip rim, apical view. (**g**) Posterior part of lip rim, lateral view. (**h**) Posterior part of lip rim, apical view; *arrow*—double peak denticle.

Nematodes were robust, tapering at both ends and white to yellowish in colour. Anterior end (Figure 1a and Figure 2a,b) exhibited a terminal triangular mouth surrounded by three prominent lips: dorsal lip with two double elliptical papillae (Figure 1b and Figure 2c,f) and two ventrolateral lips each with one double elliptical papilla and amphid adjacent to externolateral papilla (Figure 2d,g). Lips were slightly hexagonal with rounded anglěěes. Central upper rims of lips more or less inflexed (Figure 2h). The height of the lips was 200–240 (227) and the width was 220–280 (249). One ring of denticles (Figure 3) was situated on the inside of each lip edge, terminating 20–38 from the base of lip (Figure 2g arrowhead). Each lip was reinforced by labial pulp with two main branches ending and dividing anteriorly into two parts. Bifurcation was very slight. Interlabia and cuticular lateral alae were absent. Denticles were located close to each other and varied in shape and size. Denticles were mainly pyramidal at anterior and lateral edges of lips; rarely, some peaks were broadly rounded or bicuspid (Figure 3c,f,h). The height and width varied in the ranges 2.0–5.0 (3.41) and 1.5–2.8 (2.38), respectively. Denticles had double peak maximum width 4.0. Denticles on lateral sides were smaller with height 2.0–3.7 (2.73) and width 1.2–2.9 (2.37). Denticles on the distal line of the lip base were spaced closer together and had mostly rounded tops with height 1.2–2.2 (1.68) and width 1.2–1.9 (1.54). On a section of anterior margin of lip 60 wide, the number of denticles varied between 17 and 31; the number of denticles at the base of the lips (the same width) varied between 27 and 39. Cuticle was clearly transversely grooved in intervals of 5–8 (Figure 2e). Oesophagus was muscular, cylindrical, and slightly broader posteriorly than anteriorly. Excretory pore was slightly posterior to the nerve ring and stood on a slightly elevated cuticle, with an opening surrounded by circular muscle.

Male (based on two specimens): Body length 9.3–10.1 cm, maximum body width 2.40–2.50 mm. The width of the anterior extremity at the base of the lips was 458–479; the width of the posterior extremity at the level of the cloaca was 318–549. The distances of the nerve ring and the excretory pore from the anterior extremity were 1.05–1.20 mm and 2.67–3.08 mm, respectively. The tail length was 236–390, tapered conically, bearing terminal mucron 19–28 long (Figure 1d,e). There were five pairs of post-cloacal papillae, the first two pairs doubled; there was one single papilla on the upper cloacal lip (Figure 1d,e). The total number of pre-cloacal simple papillae was 64–75 pairs, formed into two longitudinal lateral rows on the sides of the body towards the cloacal opening. In the bottom part of the rows, the span between the papillae was narrow and in the anterior part of the rows, the distances between the papillae were greater. The pre-cloacal papillae end extends anteriorly 3.92–4.67 mm to the tail tip. There were two slightly curved and relatively massive spicules, equal in form and length (1.065–1.223); there were no spicular alae. Proximal ends of spicules had flat endings of width 105–169; there was a distal part with an obtuse tip of width 47–49. There was no gubernaculum.

Female (based on three juvenile specimens): Body length 11.9–19.2 cm, maximum body width 3.1–4.6 mm. The width of the anterior extremity at the base of the lips was 620–753; the width of the posterior extremity at the level of the anus was 1.07–1.19 mm. The length of the oesophagus was 6.23–7.76 mm; the maximum width was 0.98–1.03 mm. The distances of the nerve ring and the excretory pore from the anterior extremity were 1.14–2.33 mm and 1.46–2.15 mm, respectively. The vulva (Figure 1c) was in the form of a transverse slit with rounded non-prominent lips, situated at about the first third of the body. The vagina separates into two posteriorly directed uteri just posterior to the vulva. The tail (700–978 long) tapered conically (Figure 1f), with mucron 52–69 long. The distance of the phasmids from the posterior extremity was 294–339. Eggs were not developed.

### 3.2. Molecular-Genetic Analysis

#### Characterisation of 18S rDNA Region

Two partial 18S rDNA sequences (LN600406 (*n* = 2; 843 bp) and LN600407 (*n* = 3; 839 bp)) were obtained for *A. lumbricoides* isolated from *P. abelii* in Sumatra. Both sequences are almost identical, but LN600407 has a heterozygote site (S, G/C) in the position of 457. No interspecies genetic variation among 18S rDNA sequences generated in the present study was found (the identical 18S rDNA sequences were also obtained from pigs’ ascarids (*A. lumbricoides*–access. no. LN600408) and from ascarid parasitising the striped skunk in the Czech Republic (*B. columnaris*–access. no. LN600409)). The sequence comparison through NCBI BLAST (February 2023) showed 100% identity with three sequences of *A. suum* (MN558962 (dog; China), AF036587 (host not stated) and U94367 (pig; USA)), one sequence of *Ascaris* sp. (JN256985 (hoolock gibbon; China), two sequences of *B. procyonis* (U94368 (raccoon; USA), and KC172105 (raccoon dog; Norway)).

### 3.3. Characterisation of ITS Region

No intra-individual variation for the *ITS* region was detected in all five *Ascaris* specimens isolated from *P. abelii* in Sumatra. The resulting *ITS* sequence (ID OQ539679) had a total length of 1080 bp (the lengths of the partial *18S*, complete *ITS1–5.8S–ITS2* and partial *28S* sequences were detected in positions 1–145 bp, 146–593 bp, 594–750 bp, 751–1024 bp, and 1024–1080, respectively). BLASTn search (February 2023) revealed 100% identity with two sequences of the whole *ITS* region (LC422642, LC422643) obtained from *A. lumbricoides* parasitising humans in Japan, whereas the *ITS1* sequence (diagnostic region) corresponded to the previously characterised genotype G1 (AJ554036 [64]), which is the most prevalent *Ascaris ITS1* genotype in humans [64,65]. For the *5.8S* and *ITS2* region, no informative nucleotide differences were detected.

### 3.4. Characterisation of CO1 mtDNA Region

One *CO1* sequence variant (LN600399; 441 bp) was obtained from five specimens of *A. lumbricoides* isolated from *P. abelii* in Sumatra. BLASTn search (February 2023) revealed 100% identity with the sequences representing the most frequent *CO1* haplotype of *A. lumbricoides*/*suum* parasitising humans and pigs (also indicated in dog; Mohd-Shaharuddin unpublished) with worldwide distribution (named HapA1 in the present study; H12 in [49]; H1 in [48]; H29 in [51]; and US4 in [50]; for detailed information see Table 3).

### 3.5. Phylogenetic Analyses

**Figure 4:**
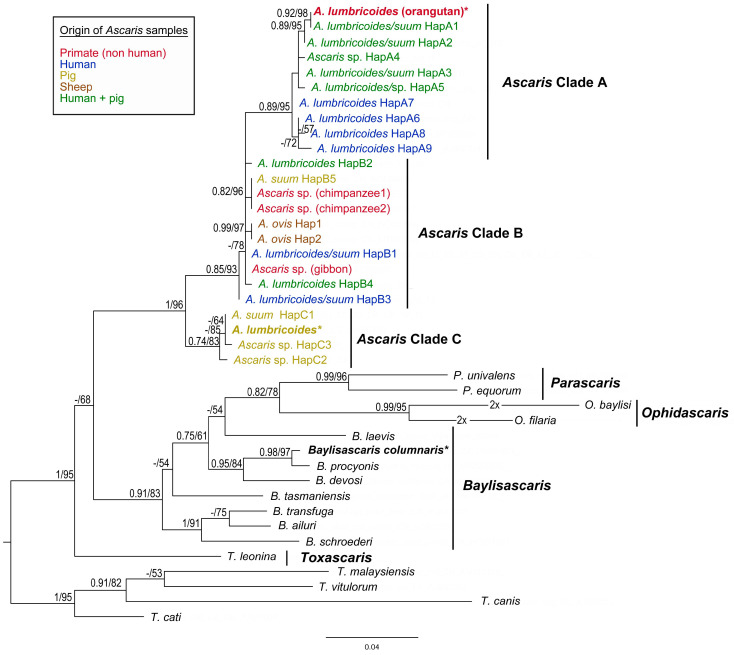
Maximum likelihood (ML) tree of Ascarididae species generated from partial *CO1* mtDNA sequence alignment. Values along the nodes indicate posterior probabilities from BI and bootstrap values from ML analyses. Dashes indicate values below 0.70 and 50, respectively. Sequences obtained in the present study are shown in bold and highlighted with asterisks. Host origin of the *CO1* haplotypes of *Ascaris* species is marked by different colours.

**Figure 5:**
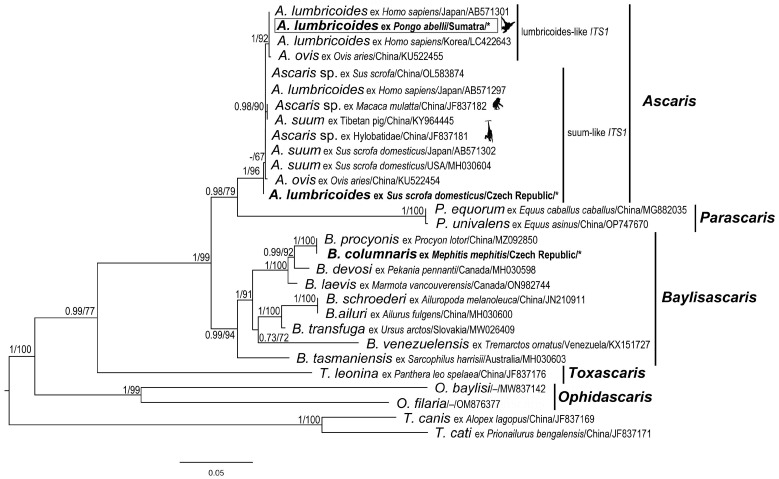
Maximum likelihood (ML) tree of Ascarididae species generated from complete *ITS* (*ITS1 –5.8S–ITS2*) sequence alignment. Values along the nodes indicate posterior probabilities from BI and bootstrap values from ML analyses. Dashes indicate values below 0.70 and 50, respectively. Sequences obtained in the present study are shown in bold and marked with an asterisk. The sequence from orangutan host in this study is in the rectangle.

The tree topologies obtained through BI and ML phylogenetic analyses of both datasets (*CO1* and *ITS*) were almost identical, and the phylogenetic reconstructions inferred from ML analyses are shown in Figure 4 and Figure 5.

The final alignment of *CO1* sequences yields 384 characters (117 variable, 88 parsimony-informative). Results of the present phylogenetic analyses based on *CO1* sequences are congruent with the previous phylogenetic studies of *Ascaris* species [47,48,66,67]. The *CO1* haplotypes of *A. lumbricoides*/*suum* form three main clades (A, B and C), with clade C being basal to the two other clusters (Figure 4). The sequence of *A. lumbricoides* from *P. abelii* obtained in the present study clusters together with *CO1* haplotypes of clade A. The *CO1* sequence of *A. lumbricoides* isolated from domestic pig in Czech Republic belonged to clade C and it is identical as the most frequent “pig” haplotype (named HapC1 in the present study, H64 in [48], H1 in [49], and H3 in [51]) with distribution in Europe and Africa.

The final alignment of *ITS* sequences yielded 857 characters (453 variable, 309 parsimony-informative). The resulting phylogenetic tree based on the *ITS* sequences is shown in Figure 5. The *ITS* sequence (orangutan) obtained in the present study from orangutans was nested within the lumbricoides-like ITS1 clade, which was highly supported by both analyses (PP = 1; BS = 92%).

## 4. Discussion

It was difficult to identify the agents of *Ascaris* infections among primates with the species level before [19,68]. In this research, it was possible to establish the identity of ascarids from Sumatran orangutans by means of a morphological and metrical study. Moreover, some details (anterior extremity) using SEM were also determined and compared with descriptions of *A. lumbricoides* from man [68,69,70,71].

Although the number of samples studied might seem very low for description, in practice, even when hosts are massively parasitised, they excrete huge numbers of parasite eggs, but the detected presence of adult worms is very sporadic in faecal samples. Therefore, the adult ascarids found in the present study represent a significant and rare finding. Up to date, the last documented record of nematode adults shed by orangutans living in the native locality date was in 1877 [28]. It is also obvious that the infection was really massive because five worms from two hosts were obtained in a very short period (one year of a 20-year long-term study focusing on orangutan parasites and natural antiparasitic drugs).

Former findings and the determinations of ascarids in orangutans are not accompanied by information on their morphometry except length of bodies at *A. satyri* by Chatin [28] and *A. lumbricoides* by Canavan [30]. Canavan reported *A. lumbricoides* from the Bornean orangutan, describing one male with a body length of 144 mm and two females with body lengths of 148 mm and 165 mm [30]. Many authorities consider *A. lumbricoides* and *A. suum* Goeze, 1782, to be morphometrically indistinguishable [72,73,74]. On the other hand, several authors found a specific morphological point of difference when studying the rows of denticles in these species’ mouths ending at the lips [75,76,77,78]. Others documented the great variability in the shape and form of the denticles [79,80,81,82]. Based on the morphology of the denticles documented via SEM, present results were compared with data on measurements of previously mentioned authors. So far, only Hartwich reported that the denticles of *A. lumbricoides* in the upper-centre of the lips reach a height of 2–7 µm and a width of 2–6 µm [69]. Borkovcová found the heights and widths of all forms of denticles in the lips of *A. lumbricoides* to be in the range of 0.7–4.2 µm and 1.3–2.7 µm, respectively [80]. The dimensions of the denticles of ascarids in this study material from Sumatra (height range: 1.2–5.0; width range: 1.2–2.9) correspond with data in Borkovcová [80].

The main features for differentiating and determining morphospecies of *A. lumbricoides* established by previously mentioned authors are as follows: (1) the shape (morphology) of pulps of lips, the anterior lobes of which are entirely divided; (2) males possessing five pairs of post-cloacal papillae, the first two pairs (located closest to the cloaca) being doubles. This feature was already mentioned by Schneider [83] and confirmed by Baylis and Daubney [29]. Using an SEM study, Uni and Takada also documented the number and distribution of papillae, including phasmidial openings situated between the second (double) and third (simple) post-cloacal papillae [84]; (3) the proximal ends of spicules are consistently straight; (4) in females, the vulva is located at the beginning of the second third of the body length (the vulva divides the body in the ratio range 1:2.0–1:2.1). A comparison of the main values characterising morphospecies of *A. lumbricoides* can be seen in Table 4.

In the opinion of Sprent, the inadequacy of the description of the species *A. satyri* prevents its inclusion in the genus *Ascaris* [19,77]. After studying the original description, authors of the presented work do not agree with this opinion and suggest considering it as species inquirenda. According to Chatin’s description without species illustrations [28], males do not exceed 90–100 mm in length, while females have an average body length of 120 mm; the colour is milk white, or slightly yellowish; the transverse cuticle striation and layer of subcutaneous musculature are thick; the description of the digestive system refers to the shape of the oral opening with three lips (the presence of denticles on the edge of the lips) and the shape of the oesophagus. Chatin also stresses that the eggs (elliptical in shape, 0.069 mm) are smaller than those of *A. lumbricoides* [28]. Even if these features do not differentiate it from *A. lumbricoides* this taxon should clearly belong to the genus *Ascaris*.

For the differentiation of *A. satyri* females in connection with the opinion of Blanchard [27], Chatin states the morphology of the reproductive organs to be the determining feature [28]. According to Baylis and Daubney, the taxonomic importance of this feature is small [29]. This opinion is generally accepted and thus this feature cannot be used to differentiate these species. It needs to be also noted that the taxon *A. satyri* is not morphologically or molecularly distinguishable from *A. lumbricoides* and it *A. satyri* is classified as a doubtful species with inadequate description.

Several approaches in molecular biology have been used before now with the aim of distinguishing ascarids to the species level (e.g., PCR-RFLP, sequencing of PCR products of selected DNA markers, microsatellites polymorphism, completion of the mitochondrial genome, NGS) [14,65,66,85,86,87]. In this work, ascarids from Sumatran orangutans were studied at the routine molecular level for the first time.

Even though the *18S* is very often considered to be good and provable barcoding marker for species identification, it could not be used for comparison or phylogenetic analyses due to the very low variability inside the genus *Ascaris*. It is proved to be not suitable for identification of ascaridoid nematodes and for the phylogeny of low level taxa. Null or very low variability in the *18S* region confirms the claims of Li, Blaxter and their colleagues [88,89] that rDNA cannot provide entirely satisfactory solutions for species and genus classification (talking about the genera *Toxocara* and *Baylisascaris*), mainly because rDNA contains fewer informative sites and is less useful at a lower level. Presented findings in *Ascaris* confirmed this stage. Two obtained *18S* ascarid variants from orangutan hosts differed only by one SNP (mixed base S in position 547 in LN600407) and no other variability was observed even between ascarids from different hosts (domestic pig and stripped skunk). The variant containing pure cytosine in this position was not detected.

Concerning the *ITS* region, *ITS2* and *5.8S* are highly conserved inside the *Ascaris* spp. and therefore are considered to be unsuitable markers for distinguishing *Ascaris* species. The *ITS2* was regarded as not informative for differentiating between two species in the past [51]. However, inside the *ITS1* region, considered to be the suitable diagnostic region for distinguishing *A. suum* and *A. lumbricoides*, two from six diagnostic sites (indicated in the past [64,90]) were demonstrated in our study. Differences at positions 274 (G/C) and 389 (T/A) (ID OQ539679) seem to be the most usable to distinguish between the species (*lumbricoides*/*suum*) or better lumbricoides-like and suum-like variants. The observed variability in the number of T nucleotide repeats at position 262 (ID OQ539679)—8–14 bases in *Ascaris* spp., 4 bases in *Baylisascaris*—was not considered to be an infallible diagnostic variability. This variability appears to be strongly affected by sequencing discrepancies; in this study, it was even necessary to use internal primers to obtain reliable sequences of this region of *ITS1*. According to the present findings in *ITS1* variability, all samples from Sumatran orangutans were identified as *A. lumbricoides* representing the G1 genotype of lumbricoides-like ITS1 clade characterised by Peng and colleagues [64]. To date, it has been identified mainly from humans in Asia (Iraq, Korea, China, India, Bangladesh, Laos, Myanmar, South Korea, Thailand, Japan) but also as a dominant haplotype from humans in Brazil [65]. The second clade, suum-like ITS1, is characterised well by the C and A nucleotides in the aforementioned positions, respectively. Moreover, the obtained *ITS* variant from pigs in this study (ID OQ539680) is unique, confirmed in two specimens, and not described before. It is well characterised and distinguishable from other pig variants by CG deletion in the *ITS2* region (observable in position 909–910 bp in ID OQ539679 and in other isolates from pigs), by noticeably longer T-nucleotide repetition (14 bases at position 102, ID OQ539680), and more unique SNPs compared to the other sequences from suum-like *Ascaris* sequences (position in ID OQ539680-nucleotide: 51-T, 101-C, 647-C).

Concerning the variability of *CO1* sequences in the database, a huge range of those defined as *A. suum* or *A. lumbricoides* are 100% identical and no interspecific differences are evident even in validated reliable genetic markers. After the sequencing of the complete mitochondrial genomes of *A. lumbricoides* and *A. suum* from China, very little divergence was found comparing them*:* 1.9% in mitochondrial genomes of Chinese isolates of these two species and 1.5% comparing isolates of *A. suum* from China and the USA [88,91]. *CO1* marker is used frequently, and it appears to be a good candidate for the identification of genera, and with a reasonable degree of stringency, even for the distinguishing of species.

The present phylogenetic findings highly correspond with the previous reports [47,48,66,92,93,94]. *Ascaris lumbricoides* isolated from Sumatran orangutans showed a very close relationship to the human genotypes representing the most frequent lumbricoides-like genotypes worldwide (HapA1 for *CO1*, G1 for *ITS1*). For a comparison of ape host effects (*Pan troglodytes* Blumenbach, 1775, *Hylobates hoolock* Harlan, 1834, *Macaca mulatta* Zimmermann, 1780, *Pongo abelii)*, several different sequences were applied for phylogenetic analysis. Relationships with other isolates from non-human primates (chimpanzees, gibbons) were not as close as one could expect. The *CO1* sequences from chimpanzees’ ascarids were identical to sequences obtained from Ascaris parasitising pigs in China (marked as HapB5). In gibbons, the most frequent haplotype HapB1 was identified. It occurs very often in humans and pigs, and its distribution is also very wide (Table 3). Two non-human primates’ *Ascaris* sequences were included also in the *ITS* region analysis. Meanwhile, concerning Sumatran orangutans linked to lumbricoides-like genotypes, the sequence from gibbon parasites was identical with G3 *ITS1* genotype (JF837181) according to Peng and colleagues [64]. The one from macaque (JF837182) was also in a suum-like ITS1 clade with little sequence differences (indels) compared to the G3 genotype.

The present study on Sumatran ascarids brings a hint of evidence that *A. lumbricoides* and *A. suum* can be (partially) genetically distinguished. According to present results on *ITS* and *CO1* variabilities and presented molecular phylogenetic findings, the *Ascaris* species should be, for now, primarily divided into two main genotypes: lumbricoides-like genotype and suum-like genotype, or possibly human-like and pig-like types suggested by Zhou and colleagues [87]. However, the trend of host-based naming (*lumbricoides*/*suum*/*ovis*) should finally be abandoned as it is misleading. The present results support the conclusions of Leles and others [95], who formally synonymised *A. suum* as a junior name of *A. lumbricoides*. Simply *Ascaris lumbricoides* should be considered the valid name. All possible discrepancies observable in the found ascarid genotypes can result from host switching of parasite and hybridisation, leading to forming hybrid genotypes [72,87,96].

Future perspectives should lead to combined analyses of concatenated datasets of especially both of the provable genetic markers for *Ascaris* species—*ITS1*, *CO1*—and possibly others [93]. Only thus can it be confirmed that lumbricoides-like genotypes may be represented by CO1 clades A and B, and suum-like genotype may be represented by the CO1 Ascaris clade C and by corresponding *ITS* genotypes, respectively. Moreover, the detailed morphological descriptions of these species should be performed firstly, and the diagnostic features should be defined well. Morphological and molecular diagnostics would be performed in combined matrix analyses. Only after this will it be possible to give inevitable proof of distinguishing *A. lumbricoides* and *A. suum* at a species level.

## Figures and Tables

**Table 2:** List of primers used for PCR amplification of mitochondrial and nuclear markers in the present study.

Locus	Primer Name	Direction	Sequence (5′-3′)	PCR Fragment Size (bp)	Ta (°C)	Reference
*18S*	18S-F_P0	Forward	CGCGAATRGCTCATTACAACAGC	>835	54 °C	[44]
	18S-R_P1	Reverse	GGGCGGTATCTGATCGCC			
*COI*	CO1-F	Forward	TGGTTTTTTGTGCATCCTGAGGTTTA	441	55 °C	[46]
	CO1-R	Reverse	AGAAAGAACGTAATGAAAATGAGCAAC			
*ITS1*	AscarisITSF1	Forward	CGAGCAGAAAAAAAAAAGTCTCC	558	55 °C	[45]
	AscarisITSR1	Reverse	GGAATGAACCCGATGGCGCAAT			
*ITS2*	ITS2F	Forward	CGAGTATCGATGAAGAACGCAGC	449	52 °C	[47]
	ITS2R	Reverse	ATATGCTTAAGTTCAGCGGG			
*ITS1-5.8S-ITS2*	TW81	Forward	GTTTCCGTAGGTGAACCTGC	>870	54 °C	[42]
	AB28	Reverse	ATATGCTTAAGTTCAGCGGGT			
	18SNemF	Forward	TTGATTACGTCCCTGCCCTTT	>1080	55 °C	[43]
	28SNemR	Reverse	GGAATCATTGCCGCTCACTTT			

Ta = annealing temperature.

**Table 3:** List of *CO1* sequences of selected representatives of Ascarididae used in the phylogenetic analyses with host species, countries of collection, and GenBank accession numbers.

Name of *CO1* Consensus/Haplotype Sequence	Host	Geographical Origin	GenBank Accession Numbers (Haplotype Identification)
*A. lumbricoides*/*suum* HapA1 (consensus)	*Homo sapiens* Linnaeus, 1758; *Sus scrofa* Linnaeus, 1758; *Canis familiaris* Linnaeus, 1758	Italy, Uganda, Zanzibar (Tanzania), Banglades, Kenya, USA, Thailand, Myanmar, Brazil, China, Malaysia, Angola, Nepal	KC455934 (cavH12); EU582484 (betH1); KY200855 (US4); MF358911; MF358930; MH800233; MH800278-282; AJ968336; MK792803-809; MK143384-390
*A. lumbricoides*/*suum* HapA2 (consensus)	*H. sapiens*; *S. scrofa*	Bangladesh, Uganda, Tanzania, Guatemala, Nepal, Thailand, Myanmar, Brazil	KF719095 (betH32); KF719112; KF719099; KF719099; KF719097; KF719096; MF358918 (sadH58); MF358909; MH800268; HM602025
*A. lumbricoides*/*suum* HapA3 (consensus)	*H. sapiens*; *S. scrofa*	Slovakia, Italy, Hungary, China, Uganda, Great Britain, Denmark, Tanzania, Guatemala, Philippines, USA, Japan, Brazil, Thailand, Laos, Myanmar	KC455927 (cavH5); AJ968334; KF536870 (betH28); KF719120; KF719122; KF719129; KF719148; KY200852 (US1); JN575629; MF358933; MF358935; MF358922; MK143379; ON725083
*Ascaris* sp. HapA4 (consensus)	*H. sapiens*; *S. scrofa*	Italy, Denmark, USA	KC455933 (cavH11); KF719139; KY200853 (US2)
*A. lumbricoides*/sp. HapA5 (consensus)	*H. sapiens*; *S. scrofa*	China, Japan	AJ968329; JN575630
*A. lumbricoides* HapA6 (consensus)	*H. sapiens*; *C. familiaris*	Bangladesh, Tanzania, Thailand, Myanmar, Malaysia	EU582491 (betH8); MF358928; MF358932, MK792800; MK792810-11
*A. lumbricoides* HapA7 (consensus)	*H. sapiens*	China, Laos, Thailand, Myanmar	AJ968333; MF358910; MF358912; MF358916
*A. lumbricoides* HapA8	*H. sapiens*	Thailand	MF358906 (sadH93)
A. lumbricoides HapA9	*H. sapiens*	China	AJ968324
***A. lumbricoides* (orangutan)**	***Pongo abelii* Lesson, 1827**	**Indonesia (Sumatra)**	**present study (LN600399)**
*A. ovis* Hap1	*Ovis aries* Linnaeus, 1758	China	NC036666
*A. ovis* Hap2	*O. aries*	China	MT993838
*A. lumbricoides*/*suum* HapB1 (consensus)	*H. sapiens*; *S. scrofa*	Italy, Zanzibar (Tanzania), Uganda, Denmark, Great Britain, Japan, USA, Brazil, China, Lithuania, Jordan, Netherlands, Slovakia, Thailand	KC455929 (cavH7); EU582490 (betH7); AB591796-98; AB591804-05; KY200854 (US3); MH674441-42; AJ968332; AJ968338; OL960110; KY368764; KY368763; KY368759; KY368756; JN575631; MW006083
*A. lumbricoides* HapB2 (consensus)	*H. sapiens*	Zanzibar (Tanzania), Uganda, Guatemala, Brazil	EU582486 (betH3); MH800259; MH800275
*A. lumbricoides*/*suum* HapB3 (consensus)	*H. sapiens*, *S. scrofa*	Zanzibar (Tanzania), China	EU582493 (betH10); AJ968340
*A. lumbricoides* HapB4 (consensus)	*H. sapiens*	Brazil	GU326953; MH800220; MH800225; MH800229; MH800237; MH800252
*A. suum* HapB5	*S. scrofa*	China	AJ968337
*Ascaris* sp. (gibbon)	*Hoolock hoolock* (Harlan, 1834)	China	KC839987
*Ascaris* sp. (chimpanzee1)	*Pan troglodytes* (Blumenbach, 1775)	China	KC839986
*Ascaris* sp. (chimpanzee2)	*P. troglodytes*	China	EU628687
*A. suum* HapC1 (consensus)	*S. scrofa*	Tanzania, Uganda, Denmark, United Kingdom, Slovakia, Italy	KF719131-34 (betH64); KC455923 (cavH1); JN575632; KY045804
*Ascaris* sp. HapC2 (consensus)	*S. scrofa*	Italy, Slovakia	KC455924 (cavH2); MZ008285 (NHap12)
*Ascaris* sp. HapC3 (consensus)	*S. scrofa*	Slovakia	KC455925 (cavH3); JN575633; MZ008286 (NHap13)
***A. lumbricoides***	***S. scrofa***	**Czech Republic**	**present study (LN600400)**
*Baylisascaris ailuri*	*Ailurus fulgens* F. Cuvier, 1825	China	HQ671080
***B. columnaris***	***Mephitis mephitis* (von Schreber, 1776)**	**Czech Republic**	**present study (LN600401)**
*B. devosi*	*Gulo gulo* (Linnaeus, 1758)	Canada	KM216983
*B. laevis*	*Marmota vancouverensis* Swarth, 1911	Canada	ON982731
*B. procyonis*	*Procyon lotor* (Linnaeus, 1758)	USA	MW385530
*B. schroederi*	*Ailuropoda melanoleuca* (David, 1869)	China	HQ671081
*B. tasmaniensis*	*Sarcophilus harrisii* (Boitard, 1841)	Australia	MH795156
*B. transfuga*	*Ursus maritimus* Phipps, 1774	China	HQ671079
*Ophidascaris baylisi*	*Python molurus bivittatus*	China	MW880927
*O. filaria*	*Elaphe carinata*	China	MH285588
*Parascaris equorum*	*Equus caballus* Linnaeus, 1758	China	MK209666
*P. univalens*	*Equus asinus* Linnaeus, 1758	China	MK209669
*Toxascaris leonina*	*C. familiaris*	Australia	AJ920063

Derived genotypes shortcuts: cav [49]; bet [48,52]; US [50]; sad [51]; NHap [53].

**Table 4:** Selected measurement of the *A. lumbricoides* (males and females) parasitising man (*Homo sapiens*) and Sumatran orangutan (*P. abelii*).

Dimensions	Male Specimens			Female Specimens		
Host	*H. sapiens*		*P. abelii*	*H. sapiens*		*P. abelii*
No. of specimens			2			3
Body length (cm)	15.0–25.0	7.0–25.0	**9.3–10.1**	25–40	8–40	**11.9–19.2**
Oesophagus length (mm)	6.0–6.5	4–10	**6.4–9.7**	6.0–6.5	6–12	**6.2–7.8**
Ratio oesophagus/body length (in %)	4.0–2.6	8.6–4.0	**9.7–7.0**	2.4–1.63	7.5–3.0	**6.52–4.42**
Tail length	-	320–700	**236–390**	-	500–2000	**700–978**
Ratio tail/body length (in %)	-	4.6–2.8	**3.9–2.5**	-	6.3–5.0	**5.5–5.0**
Number of precloacal papillae	70	40–75	**64–75**	-	-	**-**
Spiculae length	2.0	930–2360	**1065–1223**	-	-	**-**
Vulva distance (mm)	-	-	-	-	30–135	**42–65**
Ratio vulva distance/body length (in %)	-	-	-	-	27.7–29.6	**28.3–29.5**
References	[71]	[69]	**Present study**	[71]	[69]	**Present study**

## Data Availability

The datasets supporting the conclusions of this article are included within the article (and its additional files). DNA sequences generated and analysed during the current study are available in GenBank repository under accession numbers.
